# Improving Communication and Management Following a Positive Home HPV Self-Sampling Result: Comparing Intervention Strategies Between the HOME and STEP Trials

**DOI:** 10.1177/26884844251371093

**Published:** 2025-08-25

**Authors:** Jasmin A. Tiro, Meera Muthukrishnan, Sadie Metcalfe, Kris Hansen, John Lin, Caitlin N. Dorsey, Hongyuan Gao, Catherine Lacey, Melissa L. Anderson, Richard T. Meenan, Beverly B. Green, Angela Sparks, Rachel L. Winer

**Affiliations:** ^1^ Department of Public Health Sciences, University of Chicago, Biological Sciences Division, Chicago, Illinois, USA.; ^2^ Division of Public Health Sciences, Department of Surgery, Washington University School of Medicine, St. Louis, Missouri, USA.; ^3^ Kaiser Permanente Washington Health Research Institute, Seattle, Washington, USA.; ^4^ Department of Epidemiology, University of Washington School of Public Health, Seattle, Washington, USA.; ^5^ Center for Health Research, Kaiser Permanente Northwest, Portland, Oregon, USA.; ^6^ Department of Health Systems Science, Kaiser Permanente Bernard J. Tyson School of Medicine, Pasadena, California, USA.; ^7^ Washington Permanente Medical Group, Seattle, Washington, USA.

**Keywords:** abnormal test results, cervical cancer screening, HPV self-sampling

## Abstract

**Background:**

Mailed human papillomavirus (HPV) self-sampling kits improve cervical cancer screening adherence. The HOME trial found information needs and anxiety among HPV-positive patients. We designed a STEP trial to test optimized intervention strategies with bolstered educational materials and a centralized nurse communicating positive results. Here, we evaluate the effect of the strategies by comparing interviews of HOME and STEP participants receiving HPV-positive results.

**Materials and Methods:**

STEP participants were interviewed during December 2021–March 2022, and asked about their kit reaction and nurse communication, and surveyed on attitudes toward the kit. Transcripts were analyzed in two phases: (1) Coders used iterative content analysis to organize codes into node reports and identify themes and (2) coders compared node reports between the HOME and STEP trials.

**Results:**

Sociodemographic of 46 HOME and 28 STEP participants were similar (White, older, had prior Pap). Participants from both trials appreciated the kit’s convenience, although some questioned its accuracy compared to clinician-performed screening. While many STEP participants were surprised by the positive result, most felt reassured by the nurse and understood the recommended follow-up. STEP participants expressed fewer negative emotions. More STEP than HOME participants believed the HPV result was correct (86% vs. 59%) and trusted it (90% vs. 65%). Willingness to recommend the HPV kit to a friend and use it in the future was high in both the trials.

**Discussion:**

Qualitative comparison of HOME and STEP participants’ reactions suggests STEP patients received the information needed to understand HPV-positive results and complete follow-up. Findings support a centralized nurse communicating results and building trust in this new screening technology.

## Contributions to the Literature

- This qualitative analysis of interviews investigates different intervention strategies for human papillomavirus (HPV) test result communication by a primary care provider or centralized nurse.
- Patients reported fewer informational needs, understood next steps in the screening process, and felt reassured after communicating with the nurse.
- A centralized nurse providing consistent communication may be key to ensure trust in HPV self-sampling kits and adherence to recommended follow-up.
- Future studies should evaluate other models for communicating positive results suitable for lower-resource settings.

## Reporting Standards

The authors used the COREQ checklist to assess the complete and transparent reporting of the qualitative interviews (see Supplementary Appendix A2).

## Background

Almost 14,000 cervical cancers are diagnosed annually in the United States.^1^ More than half of cancers occur in individuals with a cervix who are under-screened.^2,3^ Current screening guidelines recommend that individuals with a cervix complete a Pap test every 3 years or high-risk human papillomavirus (hrHPV) testing, either alone or with a Pap (*i.e.,* co-test), every 5 years.^4^ Screening declined from 86% in 2005 to 73% in 2021^5^ with COVID-19 pandemic significantly impacting the delivery of screening.^6^ Under-screened groups face logistical barriers, such as no source of regular care, competing demands, lack of childcare, and scheduling difficulties.^7^ Recently approved by the US Food and Drug Administration^8^ for use in clinics, human papillomavirus (HPV) self-sampling (HPV-SS) kits offer a promising solution to these barriers. While only very recently included in some US screening guidelines, these kits have the potential to significantly improve screening rates and address barriers.^9–11^

Meta-analyses of randomized controlled trials showed that HPV-SS increases screening uptake.^12^ Most of these trials mailed the HPV-SS kit and were conducted in Europe and Australia. The only US-based trial, the HOME trial (2014–2017),^13^ compared the effectiveness of mailing HPV-SS kits to usual care in patients who were overdue for screening. HOME-used quantitative methods to examine clinical and screening outcomes and qualitative interviews to explore patient experiences following a positive HPV kit result. The HOME trial found that 26% of the intervention group got screened (vs. 17% in the usual care group) with suboptimal follow-up adherence (only 59% with HPV 16/18+ results [*n* = 34] completed a colposcopy and 74% with other high-risk HPV+ results had an in-clinic Pap [*n* = 102]). Several trial participants with a positive kit result reported unmet informational needs about the recommended diagnostic procedures and felt anxious about the HPV kit results.^13,14^

Based on findings in the HOME trial, we designed the STEP trial to evaluate optimized intervention strategies for (1) educating patients about the screening process and the importance of follow-up if the HPV-SS kit is positive (print materials and a website) sent with the mailed kit invitation, and (2) explaining kit results and recommended follow-up by a centralized population health management nurse (vs. patient’s primary care provider).^15^ Delivery of kit results through a centralized nurse ensures standard, consistent, and equitable care among all patients.^16^ It is possible that not all clinicians may have received the same levels of training in communicating screening results, which may leave some patients without a full understanding of their results and/or the guideline-recommended follow-up care they need.^17^ Because of the novelty of primary HPV testing, some clinicians may lack experience in effectively communicating HPV test results. This can make it challenging to alleviate patient fears and worries about an HPV-positive result, potentially not delivering guideline-concordant messages. As a result, patients may experience distrust in and confusion with new screening technologies and health care systems.

Both the HOME and STEP trials included a qualitative investigation to assess patients’ understanding of their positive kit results. The current study analyzed participant interviews from both trials to evaluate whether the revised strategies used in the STEP trial addressed patients’ information needs, decreased negative emotions, and whether the nurse communication was perceived as helpful.

## Methods

The HOME and STEP trials were pragmatic randomized controlled trials that evaluated cervical cancer screening uptake by mailing HPV self-sampling kits to eligible patients. Eligibility criteria differed between the two trials: HOME offered kits to patients overdue for screening, whereas STEP invited three different screening history groups—overdue, due after being previously screening adherent, and individuals with unknown screening histories—and tested different interventions in arms randomized by screening history. The STEP trial aimed to build on lessons learned from the HOME trial by addressing information gaps about the necessary diagnostic procedures following a positive HPV kit result. The STEP trial updated print materials sent with the kit and sent a link to a website with the kit results. Both the HOME and the STEP trials were approved by the Kaiser Permanente Washington (KPWA) institutional review board. Details and main findings of the STEP trial (NCT04679675) are described elsewhere.^15,18^ Briefly, STEP randomized 31,149 enrollees of KPWA who had a cervix, were 30–64 years old, and were eligible for screening. Invitations for the main trial were sent from November 2020 to January 2022 during the height of the COVID pandemic when routine in-clinic cervical cancer screening had resumed at KPWA.^15,18^ Screening uptake was 14.7%–16.8% higher in the intervention arms mailed kits compared to usual care. Completion of follow-up was 76% in the HPV 16/18+ patients needing a colposcopy (*n* = 25) and 84% in the other high-risk HPV+ patients needing an in-clinic Pap (*n* = 115)^18^; completion of follow-up Pap was significantly higher in the STEP compared to HOME trial (*p* = 0.021); completion of colposcopy was not significantly different due to small sample size.

Mirroring the design of the HOME trial’s qualitative component,^14^ the STEP trial conducted semistructured interviews with a subset of the STEP trial participants who were randomized to receive an HPV-SS kit and had a positive kit result. Two intervention arms in the STEP trial were combined in this analysis; the arms differed in the kit distribution approach (“Direct-mail” arm: kit mailed after an introductory letter; “Opt-In” arm: a letter with instructions on how to request a kit by mail). In both, the letter included a brochure explaining the importance of cervical cancer screening, and the kit contained a postage-paid return envelope. Per KPWA standard protocol, laboratory staff documented kit results in the electronic health record (EHR) and released findings *via* patient portal or a mailed letter within 24 hours. The population health management nurse was notified of all positive results and called patients by the next business day to explain findings, answer questions, and schedule recommended follow-up (*e.g.,* colposcopy if HPV 16/18 positive or in-clinic cytology if positive for other non-16/18 hrHPV types only).

### Eligibility criteria and recruitment process

Interview recruitment was from December 2021 to March 2022. We queried the trial database and EHR weekly to identify participants randomized to the intervention arms who returned the kit and had a positive result. Participants were invited for the interview after 6 months to allow time to complete diagnostic follow-up (primary outcome of the STEP trial). We used the same two-stage telephone-based invitation and informed consent process as in the HOME qualitative study.^14^ Participants had no prior relationships with researchers or interviewers. Participants understood researchers’ qualifications, goals of the qualitative component of the STEP trial, and received a $50 incentive.

### Interview guide

We made minor adaptations to the HOME interview guide. Supplementary Appendix A1 highlights in yellow the changes to the guide that probed about reaction to the mailed kit, the process to request a kit, and their reactions to receiving a positive result. We included the same 8-item attitudinal survey of experiences using the kit and preferences for the future (self-sampling kit or clinician-collected test) with a 5-point Likert scale ranging from strongly disagree to strongly agree. C.N.D. conducted each interview with participants, took field notes, and audio recorded the sessions. Interview recordings were 20–50 minutes in duration; they were transcribed and anonymized before coding. Transcripts were not returned to participants for comment or correction. We did not conduct any repeat interviews with participants. J.A.T., K.H., and C.N.D. reviewed transcripts and discussed data saturation before completing data collection.

### Analytic strategy

We designed a two-phase thematic coding process, all done through Microsoft Excel. In Phase 1, five coauthors (M.M., J.A.T., R.L.W., J.L., and K.H.) concurrently applied similar codes from the HOME trial using iterative content analysis.^19^ Coders met after coding three transcripts to discuss whether any codebook revisions were needed. One code was added to document references to the COVID-19 pandemic. Themes were identified and discussed among the team using the framework approach.^20–22^

In Phase 2, coders discussed which trial elements differed and were similar between the HOME and STEP trials, as well as posited which elements might change coded data. For example, we expected no difference in how participants reacted to the kit invitation. However, we thought STEP participants may have reported more knowledge about HPV infection and Pap tests, felt at risk for cervical cancer, and were motivated to get screened due to the brochure. We also expected less heterogeneity in participants’ description of information needs and anxiety after the result communication because it was centrally managed by one nurse^15^ (vs. handled by each primary care provider and their team in the HOME trial^14^). Two coders (M.M. and J.A.T.) created a table to organize and juxtapose coding data from the trials, and then the group discussed whether the data were different or similar as posited. This planned comparison between HOME and STEP codes and themes helped evaluate whether the refined strategies worked as intended. Only one author (J.A.T.) participated in coding HOME and STEP qualitative data, while two other authors (R.L.W. and J.L.) participated in the interpretation of both. Participants were not involved in the coding process or theme identification.

We also calculated the mean score for the 8-item attitudinal survey (range 1–5 with higher scores indicating stronger agreement with the items) and percentage who “strongly agreed”/“agreed” with each survey item. We used Fisher’s exact test to compare if agreement for each item was significantly different between HOME and STEP. Supplementary Appendix A2 summarizes the COREQ checklist for how we comprehensively reported this qualitative study.

## Results

We identified 50 eligible STEP trial participants who had an HPV-SS positive result during our recruitment window. Of these, 56% (28 participants) completed an interview, 12% (6 participants) declined or were unable to complete a scheduled interview, and 32% (16 participants) were unreachable. Response rates of those participating in the interviews for the HOME and STEP trials were similar (Table 1). When comparing interview respondents to nonrespondents, interviewees were more likely to be White. In terms of sociodemographic characteristics, most participants were older, White, not Hispanic, and had a prior Pap documented in their EHR. In both HOME and STEP, interview completers had similar proportions positive for HPV 16/18 (vs. other hrHPV positive) and most completed timely follow-up (∼82%); follow-up completion was higher among the subset of qualitative participants compared to trial participants (described above). There were three differences among those participating in the qualitative interviews: (1) more HOME participants were enrolled in the health plan for at least 10 years than STEP participants (54% vs. 25%), (2) time between positive kit result and interview date was longer for STEP compared to HOME interviewees (average 10.2 vs. 4.2 months), and (3) more STEP participants had a Pap in the last 5 years compared to HOME participants (92% vs. 64%). The latter was expected as the HOME trial’s eligibility criteria focused on patients overdue for screening, while STEP included both overdue and due patients. In both trials, participants positively rated their experience using the HPV-SS kit (average scores were 4.39 in HOME and 4.57 in STEP on a 5-point scale; Table 2). In terms of individual survey items, more STEP participants (compared to HOME) believed the HPV-SS kit result was correct (86% vs. 59%; *p* = 0.020) and trusted the kit result (90% vs. 65%; *p* = 0.028). Willingness to recommend the HPV-SS kit to a friend and to use the kit in the future was high among participants in both trials (Table 2). A minority of participants reported preferring in-clinic screening (HOME: 13%, STEP: 24%).

**Table 1.** Sociodemographic and Health Care Utilization Characteristics of Trial Participants Invited to a Qualitative Interview After Receiving a Positive Human Papillomavirus Self-Sampling Kit Result, by Trial and Interview Status

Characteristic	HOME trial August 2014–March 2017		STEP trial December 2021–March 2022	
	Interview status		Interview status	
	Invited, nonrespondent, *n* = 29 (38.7%)	Interviewed, *n* = 46 (61.3%)	Invited, nonrespondent, *n* = 22 (44.0%)	Interviewed, *n* = 28 (56.0%)
Age group (years)				
30–39	5 (17.2)	5 (10.9)	7 (31.8)	8 (28.6)
40–49	7 (24.1)	7 (15.2)	9 (40.9)	6 (21.4)
50–64	17 (58.6)	34 (73.9)	6 (27.3)	14 (50.0)
Race^*^				
White	22 (75.9)	41 (89.1)	13 (59.1)	21 (75.0)
Black	2 (6.9)	2 (4.3)	3 (13.6)	3 (10.7)
Asian/Pacific Islander	4 (13.8)	0 (0)	2 (9.1)	1 (3.6)
Multiple race/other/unknown	1 (3.4)	3 (6.5)	4 (18.2)	3 (10.7)
Ethnicity				
Hispanic	1 (3.4)	3 (6.5)	1 (4.6)	2 (7.1)
Non-Hispanic	28 (96.6)	42 (91.3)	18 (81.8)	24 (85.7)
Unknown	0 (0)	1 (2.2)	3 (13.6)	2 (7.1)
Length of health plan enrollment^a^				
<5 years	11 (37.9)	7 (15.2)	14 (63.6)	14 (50.0)
≥5 to <10 years	8 (27.6)	10 (21.7)	4 (18.2)	7 (25.0)
≥10 years	10 (34.5)	25 (54.3)	4 (18.2)	7 (25.0)
Pap history documented in EHR^*^				
No prior Pap	11 (37.9)	7 (15.2)	5 (22.7)	4 (14.3)
≥1 Pap	18 (62.1)	39 (84.8)	17 (77.3)	24 (85.7)

Characteristics of patients with a prior Pap	*n* = 18	*n* = 39	*n* = 17	*n* = 24
Time since last Pap^b^				
<5 years	14 (77.8)	25 (64.1)	14 (82.3)	22 (91.7)
≥5 to <10 years	2 (11.1)	12 (30.8)	2 (11.8)	2 (8.3)
≥10 years	2 (11.1)	2 (5.1)	1 (5.9)	0 (0)
Most recent Pap result				
Normal	16 (88.9)	29 (74.4)	10 (58.8)	21 (87.5)
Abnormal (ASC-US or worse)	0 (0)	1 (2.6)	1 (5.9)	0 (0)
Unsatisfactory	0 (0)	1 (2.6)	0 (0)	0 (0)
Unknown result^c^	2 (11.1)	8 (20.5)	6 (35.3)	3 (12.5)
*Home hrHPV kit result*	*n = 29*	*n = 46*	*n = 22*	*n = 28*
HPV 16/18 positive	3 (10.3)	15 (32.6)	9 (40.9)	8 (28.6)
Other hrHPV positive	26 (89.7)	31 (67.4)	13 (59.1)	20 (71.4)
Completed timely diagnostic follow-up^d^				
Yes	24 (82.8)	38 (82.6)	18 (81.8)	23 (82.1)
No	5 (17.2)	8 (17.4)	4 (18.2)	5 (17.9)
Time between positive kit result and interview, average months (IQR)^*^	*Not applicable*	4.2 (1.9, 7.0)	*Not applicable*	10.2 (9.4, 11.4)

^*^ *p* Value for chi-square or Fisher’s exact test was <0.05.

^a^ Continuous enrollment for at least 3.4 years before randomization (allowing for gaps of up to 2 months) was an eligibility criterion for both trials.

^b^ An eligibility criterion for the trial was no Pap documented in the EHR for at least 3.4 years before randomization.

^c^ Pap performed outside of health care system and results were not documented in the EHR.

^d^ To be counted as timely diagnostic follow-up, patients enrolled in the HOME trial had to receive co-test (Pap and HPV tests) and/or colposcopy within 6 months; if biopsy findings warranted treatment, patients were allowed up to 12 months to receive excisional procedures. In contrast, timely diagnostic follow-up for the STEP trial was defined as receipt of recommended test/procedure after a positive kit result and within 6 months of the randomization date.

ASC-US, atypical squamous cells of undetermined significance; EHR, electronic health record; hrHPV, high-risk human papillomavirus; IQR, inter quartile range.

**Table 2.** Survey Results of Participants Who Completed a Qualitative Interview After Receiving a Positive Human Papillomavirus Self-Sampling Kit Result in a Pragmatic Trial

Scale score	HOME trial	STEP trial
	*N* = 46	*N* = 28
	Mean (SD)	Mean (SD)
Attitudinal survey—experience using HPV kit (8 items)	4.39 (0.55)	4.57 (0.78)

Individual items	% strongly agree/agree	% strongly agree/agree
I believe the HPV test result is correct.^*^	**58.7**	**86.2**
I trust the HPV test result.^*^	**65.2**	**89.7**
By using the HPV kit, I felt like I was taking control of my health.	82.6	100.0
Using the HPV kit is a good thing to do for my health.	89.1	96.4
I would recommend the HPV kit to a friend.	84.8	89.7
I would use the HPV kit in the future.	**91.1**	89.7
I would prefer in-clinic screening to using the home HPV kit.	13.0	24.1

^*^ Fischer’s exact test found significant differences in agreement between the HOME and STEP trials; *p* < 0.05.

HPV, human papillomavirus; SD, standard deviation.

### Findings from the thematic analysis

After comparing codes across HOME and STEP participants, we found that one original theme remained consistent: convenience of the HPV-SS home-based kit. Table 3 highlights exemplar quotes from both HOME and STEP participants illustrating where perspectives were similar or contrasted.

**Table 3.** Exemplar Quotes Representing Themes from HOME and STEP Trial Participants

HOME participants	STEP participants
**Theme 1: Convenience especially during the COVID-19 pandemic**	
“My initial thought was this is pretty convenient and that it takes minimal time, and I hate going to the doctor’s so that’s why I thought I’d try the screening kit.” participant #103130	“I loved the idea of doing it yourself. It’s something that obviously no one looks forward to going to the doctor to have this done, and if you can do it yourself at home, I thought that was fantastic … I’m actually three hours from my doctor each way, so this was incredibly helpful to me, to not have to drive for six hours to go have this test.” participant #7
“it was really easy to use and it seems like a really—to me, it seems like a private way to take a test and find out about something. It didn’t seem daunting to me, or overwhelming.” Participant #105723	“It’s really hard for me work wise to get away… so I kept putting it [the Pap] off... It just seemed so much easier for me, convenience wise, but also it was a great way to get me to actually take a test and make sure I was doing okay.” Participant #44
	“I was happy, I was like oh, I can get to do this by myself, I don’t have to actually go. Because I hated the procedure when I had one. It was very traumatizing. So to be able to do that in the comfort of my home was very convenient and comfortable for me.” Participant #45
	“It came at right at the peak of the pandemic, so I thought getting to do something at home, and not having to wait or not get into a clinic and/or go, especially during that time, was really fantastic.” Participant #20
**Theme 2: Adequate, accurate specimen collection—lack of understanding between Pap and HPV tests**	
“One person said that you have to know the right spot to test too. Which I don’t think a person at home, I mean everybody that I talked to said you need to do a Pap.” Participant #101654	“I don’t think that the procedure or the kit itself was confusing, but I personally am skeptical that I swabbed myself correctly, because it wasn’t as uncomfortable as it is in the office.” Participant #4
“Just how accurate or reliable it was. And what the fail rate, you know, the error rate was. Whether it was very reliable or whether it was likely to have false positives…” Participant #106892	“I think I would just in general trust the results of an in-office Pap smear more than the in-home test kit.” Participant #4
“So she explained to me at that time that the way they swab, they can swab in different areas and they don’t always catch it, so it was kind of that it was sent in for the HPV screening test because it was one more chance to catch everything, because every year Pap smear doesn’t necessarily catch everything.” Participant #101399	“I think there’s probably still a margin of error when I’m doing it myself versus when someone else can actually see in there when they’re doing it, you know?” Participant #13
**Theme 3: Intense affect after receiving positive kit results**	
“There was not a clear follow-up. There must be something in my messages or something. I do the online thing for My Group Health. . . . I guess there’s something in My Health, the messages or something, just vague, treated very vaguely.” Participant #10169	“A bit concerned (laughs)…I’ll be honest… I questioned the result at first. I just didn’t think it was right—it was confusing to me how I got the result I got.” Participant #7
“I received an email from Group Health that scared the sh*t out of me. ‘Uh, danger, danger, you tested positive for all high risk for pre cancer, call your Group Health person immediately.’ Pretty scary.” Participant #102702	“Someone called me within a couple of days, and we talked through it, and the course that they wanted me to take, which I did. I was never, like—I was completely taken care of the whole time. I never felt like I didn’t have someone there to talk to.” Participant #7
	“I felt very supported. I felt there was always someone who could answer a question if I had one. Everyone I worked with, when I went for my follow up, was very respectful. It was a pretty painless process. I didn’t have any complaints, when it was all said and done… it all made sense and was explained to me very clearly. So I thought it was good.” Participant #44
	“I felt—I guess it felt good to know that I had the results, and that I had done the testing. It did feel kind of empowering to be able to test.” Participant #5
**Theme 4: Information seeking**	
“On the internet I was looking up the numbers, because I didn’t understand what the numbers were. It was like the worst numbers you could get, is what it said—16 and 18 or something like that?” Participant #104186	“The internet…[I found] most of the same information that I already knew, and confirmation of what the nurse was telling me about potential latency, like strains, complications, follow-up care, and means of transmission.” Participant #13
“I looked on the internet. . . . and that scared me even more.” Participant #109695	“I: After you received your results, did you want any more information? P: Not necessarily. I just wanted to be checked out to make sure if this was really positive or not, and to kind of anticipate what the next steps were for what I had to do.” Participant #40
“In the health books that I have from Group Health, I’m sure I looked on WebMD and then talking to different people. I had a friend who had cervical cancer so I got a lot of information from her.” Participant #100436	“I: Thank you. After receiving your results, did you want any more information? P: More information? I guess when I called, I probably had questions, but mainly that was just to schedule it. So yeah, I felt like what you guys sent me made sense. So it was kind of like okay, the next step is—to me that was like yeah, that makes sense. I don’t know that I really needed more information.” Participant #44

HPV, human papillomavirus.

### Theme 1: Convenience especially during the COVID-19 pandemic

Both HOME and STEP participants appreciated the convenience of the HPV-SS kit and being able to do this test at home. Participants from both trials cited the benefit of reduced time as compared to Pap tests and the added benefit of privacy. Overall, they found the kit to be “way easier, and more comfortable and convenient” (STEP Participant #21). STEP participants also shared that they believed the HPV-SS kits were being offered as a response to the COVID-19 pandemic. Several STEP participants appreciated the fact that the kit kept them safe by eliminating the exposure to sick people in the clinic. One participant noted, “It came at right at the peak of the pandemic, so I thought getting to do something at home, and not having to wait or not get into a clinic and/or go, especially during that time, was really fantastic” (STEP Participant #20). Experience with self-administered COVID tests also appeared to increase comfort with self-sampling; however, concerns remained about performing the test correctly.

### Theme 2: Adequate, accurate specimen collection—continued lack of understanding between Pap test and HPV-SS kits

Participants from both trials expressed concerns about the accuracy of the HPV-SS kit results, primarily worried about whether they were doing the self-sampling “right.”

I personally am skeptical that I swabbed myself correctly, because it wasn’t as uncomfortable as it is in the office, and I know they use like a brush in the office rather than just like a Q-tip. So, to me, I would still want to go in for a regular Pap smear, because it just seemed like the difference between the two procedures was like—the gap is vast… I think I would just in general trust the results of an in-office Pap smear more than the in-home test kit.* (STEP Participant #4)*

Patients associated the discomfort and pain of collecting a cervical specimen during a Pap test with the accuracy of the sampling and validity of the results. This connection suggests that some patients still did not understand the difference between the two tests (Pap collects cervical cells to identify abnormalities, whereas an HPV-SS kit collects vaginal cells to determine the presence of an HPV infection). As a result, patients continued to worry about the accuracy of HPV-SS kit results, comparing it to the experience of receiving screening performed by a clinician.

### Theme 3: Intense affect after receiving positive kit results (surprise, anxiety, worry)

Though STEP patients still experienced surprise, fear, or confusion after receiving a positive kit result, they felt more supported during initial results communication compared to HOME participants. While surprised, patients expressed positive feelings regarding the results communication.

I will say that the whole kit process was really straightforward. I did the kit, I sent it in, I got a response, I got the positive. I knew immediately, I was told [by the nurse] what to do next. I followed through, I went and did it, and then the process was complete. So that piece actually went really smoothly for me, so I was pleased with that… which was really nice, since I was a little surprised with [the positive result]. *(STEP Participant #8)*

STEP participants felt very supported during the screening process:

Someone called me within a couple of days, and we talked through it, and the course that they wanted me to take, which I did. I was never, like—I was completely taken care of the whole time. I never felt like I didn’t have someone there to talk to. *(STEP Participant #7)*

Many STEP participants reported feeling reassured after talking with the centralized nurse who explained the results and understood the next steps in the diagnosis and management process. This feeling of reassurance was not expressed by HOME participants. Instead, HOME participants described their provider’s communication after a positive kit result as very “vague,” leaving them mostly fearful.

It was just scary, because they were saying I was positive. A possible link or cause of cervical cancer. *(HOME Participant #100436)*

### Theme 4: Information-seeking and follow-up with a provider

Though an educational pamphlet was provided with the HPV-SS kit, most participants did not recall receiving it or using it to understand their kit results. A few STEP participants sought further information on the internet, whereas others felt the communication with the nurse provided enough information that they did not need to seek information elsewhere.

Most of the same information that I already knew, and confirmation of what the nurse was telling me about potential latency, like strains, complications, follow up care, and means of transmission. *(STEP Participant #13)*

This differed substantially from the HOME trial, in which many participants also used the internet to understand what the different HPV types (*e.g.,* HPV 16 and 18) and a positive result meant.

I actually googled it, so, yeah, I guess I was a little bit confused. Yeah, I was—it was confusing, whether it was urgent or not urgent, and whether it was scary or not scary. Yeah, I felt like I needed more information. . . . *(HOME Participant #109211)*

## Discussion

While both the HOME and STEP pragmatic trials sought to boost cervical cancer screening rates by mailing HPV-SS kits to patients, their intervention strategies for patient education and results communication had a differing impact on participants’ reactions to their positive kit result. Semistructured interviews with HOME participants revealed gaps in education and information about the follow-up procedures in the event of a positive result.^14^ The STEP trial was built to address these gaps by bolstering educational materials (use of updated print materials and an informative website) and a centrally supported strategy to communicate about HPV-positive results (use of a nurse to explain findings and follow-up procedures with HPV-positive women). To our knowledge, this is the first qualitative study comparing two strategies for increasing use of and reaction to mailed HPV-SS kits.

The major consistency between the trials was the patients’ emphasis on the mailed HPV-SS kit’s convenience as compared to an in-clinic Pap test. This is consistent with Camara et al.’s systematic review.^23^ Many studies have documented barriers to completing in-clinic screening, such as lack of time due to competing demands, embarrassment about the procedure, and poor access to care.^14,24^ The convenience of the HPV-SS kit incentivizes cervical cancer screening among under-screened populations.^23–25^

Both quantitative data reported in the main trials^13,18^ and qualitative data above support that the improvements made to the communication of positive kit results in the STEP trial were effective, especially the inclusion of the centralized nurse. STEP patients reported less anxiety, fear, or surprise when receiving a positive result because they were met with guidance from the nurse (*i.e.,* explaining results and follow-up procedures, answering questions, scheduling appointments). Our findings reveal how the availability of a trained and practiced nurse can help reduce anxiety about HPV-positive results and encourage adherence to follow-up appointments. The centralization of kit-result communication is essential to maintaining patient trust and managing patients’ concerns and emotions. Centralization also has the potential to increase access to follow-up care, eliminate redundancies, reduce errors in or failures to communicate screening results, and optimize workflow in health care settings.^17,26–28^

Additionally, the presence of the centralized nurse reduced patients’ need to seek information from other sources. Some patients indicated they did not seek additional information because they were reassured by the nurse and were more focused on seeing their primary care provider to ascertain next steps. This signals trust in their clinician and desire to check in with them about clinical recommendations made by the health care system. Building trust and comfort in new screening technologies depends in part on trust in clinicians and health care systems,^23^ and is critical to preserve the patient–clinician relationship, as noted by Wood.^29,30^ The continuity in care and the effectiveness of closed-loop communication between the nurse and the patient assuaged patient fears about being HPV-positive, reduced the need to seek other (potentially inaccurate) sources for health information, and established patient trust in this new screening modality and the health system.

Though inclusion of the centralized nurse in the STEP trial appeared to improve patients’ comfort with the HPV-SS kit, it does not seem that the bolstered educational materials had the same beneficial impact. In the STEP trial, doubt in the accuracy of the results persisted. Patients did not understand the difference between a clinician-administered Pap test and an HPV-SS kit. When patients did not experience the discomfort typically associated with the Pap test, they began to question whether they were using the kit correctly. If they felt that had not properly used the vaginal swab, they may also doubt the validity of results.

This misunderstanding among patient populations suggests that education, especially about the differences between the Pap test and the HPV-SS kit, needs improvement. Because the main mode of education was written correspondence (a mailed educational brochure and a website), future studies should investigate other modes of education (*e.g.,* audiovisual aid, social media awareness, and discussions with clinicians) and outreach strategies adapted to each channel. It may also be helpful to administer educational efforts at multiple timepoints throughout the screening process (*i.e.,* before kit delivery, with kit delivery, after kit receipt, at result communication, after result communication).^14^

Limitations include that both trials were conducted with the Pacific Northwest population; thus, participants were mostly White, middle-aged, commercially insured individuals with prior Pap experience. It is critical to investigate the experiences of minoritized patients (*e.g.,* LGBTQ+, BIPOC, low-income, and less educated). These experiences could inform future efforts to tailor recruitment, education, and instructional materials^31^ and how best to use a centralized nurse to engage and support minoritized patients. Another aspect potentially limiting generalizability was that the health care system covered the cost of the centralized nurse. This may be infeasible for smaller, lower-resource health care settings, so alternative strategies for results communication may need to be explored such as assigning the responsibility to patient navigators^32,33^ whose services can now be billed and reimbursed. Additionally, the patients interviewed were those who used the kits and received a positive result; thus, future research should also investigate the reactions of screeners who used the kit and received a negative result, as well as those who chose not to use the kit.

## Conclusion

Mailed HPV self-sampling kits are a convenient and well-received cervical cancer screening option, especially for population who have transportation and childcare challenges and lack time to visit clinics and be screened. Communication by centralized nurses reassured patients about their positive kit results and supported completion of the cervical screening process critical to effective cancer prevention.

## Data Availability

Data types include deidentified participant data and data codebook. To access data, requests for data must be sent to jtiro@uchicago.edu. Data will be made available with publication to researchers whose proposed use of the data has been approved for a specified purpose. Data will be made available without investigator support to researchers with adequate resources to cover the regulatory and data sharing costs and after approval of a concept proposal aligned with current data approvals and with a signed data access agreement.

## Notes

Supplementary Appendix A1

Supplementary Appendix A2

## Abbreviations Used

EHR: electronic health record
HOME: Home-based Options to Make cervical cancer screening Easy
HPV-SS: human papillomavirus self-sampling
hrHPV: high-risk human papillomavirus
STEP: Self-Testing options in the Era for Primary HPV screening for cervical cancer
