# Supplementary material for: Improving Communication and Management Following a Positive Home HPV Self-Sampling Result: Comparing Intervention Strategies Between the HOME and STEP Trials
Source: Womens Health Rep (New Rochelle). 2025 Aug 25;6(1):771–81. doi: 10.1177/26884844251371093 (PMC12415163; doi:10.1177/26884844251371093)
Supplement: Supplementary Appendix A1 [file 26884844251371093_supp_appendixa1.docx]

**Appendix 1. Comparison of HOME and STEP Trial Semi-Structured Interview Guides**

| **HOME Trial** | **STEP Trial** |
| --- | --- |
| **Introduction & Verbal Informed Consent**  “Thank you for talking with me about your experience with the home HPV Screening Kit. We hope that interviewing women will help us learn more about this type of cervical cancer screening. The first few questions are about HPV or human papillomavirus and the new home HPV Screening Kit. We want to know what you think about the test and any interactions that you may have had with your providers about this test. Your answers will be kept confidential and will not be shared with your health care team although we may give them general feedback from the study that does not contain any personal identifiers. You can skip any questions you don’t want to answer. You can also end the interview at any time if you want to.  We would like to record this interview. We will listen to the recording, write down what you said, and then erase the recording when we are done with the study. Anything you say that could identify you will not be written down. Is this okay with you?”  *If NO, end call and thank participant for their time;*  *If YES, continue with interview* | **Introduction & Verbal Informed Consent**  Ok, before we proceed with the interview, I’m going to provide an explanation of your rights as a study participant and then ask for your consent.  The study is being done by researchers at Kaiser Permanente Washington Health Research Institute, the University of Washington, and the University of Texas Southwestern Medical Center. All of our researchers and study staff are specially trained to keep your information private. We will not use your name in any study reports. It is possible that someone other than study staff could find out you are in the study or see your private study information. Our study team will take precautions to reduce the risk of this happening. All information collected for this study will be kept confidential as provided by law.  Our study information may be used for future studies. We will remove your name and any identifying information and will not ask your additional permission to share study data for research purposes.  There are no costs to you for participating in the interview except for your time.  Being in this study may not help you personally, however, you will help us learn about experiences with self- collected human papilloma virus testing, which may improve cervical cancer screening options in the future.  Answering the survey questions is up to you. You might feel uncomfortable answering some of the questions about cervical cancer screening. You can skip any questions you don’t want to answer or stop the interview at any time.  Your decision whether to be a part of the study will not affect the health care you receive or the benefits you are entitled to.  Do you have any questions?  *IF YES, address any questions the participant has, then proceed to Interview*  *IF NO, proceed to Interview*  OK, let’s get started with the interview. |
| **General Thoughts about HPV & the Home HPV Test**  1. Tell me in your own words, what is Human Papillomavirus, or HPV?  2. Tell me in your own words what you know about the home HPV Screening Kit?  3. What do you understand it is supposed to do?  4. When you received the home HPV kit in the mail, what were your thoughts?  5. Did you talk with anyone about the kit?  6. Why did you decide to return the home HPV screening kit? [If woman states she did NOT return the kit, thank her for her time and end the call. If reported kit was returned, then continue] | **General Thoughts about HPV & the Home HPV Test**  *(Goal: Gather vernacular patients use to describe HPV and the home HPV test; elicit reaction to receiving mailed outreach invitation.)*   1. Can you tell me in your own words what Human Papillomavirus or HPV is? 2. Can you tell me in your own words what the home HPV Screening Kit is intended to do? 3. Do you remember the information you received in the mail about the home HPV kit? What were your thoughts? [*Generic probe to see if they say they returned the kit or had to request the kit:* We have been exploring different ways of distributing the kit. Did you receive the kit in the mail or did you have to request the kit by calling a number or going to a website?]    1. *If they requested the kit:* Was requesting a kit easy? Any suggestions on how to make the request process easier? 4. Once you got the kit, was it clear how to use the kit?    1. *If NO*: How can we improve the instructions?    2. *If YES*: Was it clear how to package it up and send it back? (*If not clear*: how can we explain that better?) Was it clear what would happen after the lab processed the kit and how you would get your results? (*If not clear*: what would you recommend we change?) 5. We are interested in understanding why you decided to use the home HPV screening kit. Can you tell me a little about what led you to use the screening kit instead of coming into the clinic for screening? 6. Do you remember receiving an educational brochure about cervical cancer screening in the mail before you got the home screening kit?    1. *If YES*: What do you remember about the educational brochure? [*Probes*: Can you tell me what you liked or didn’t like about the brochure? What didn’t you like? Did you learn anything about HPV or cervical cancer that you didn’t already know? What did you learn?] |
| **Quantitative Measure of Attitudes toward Home HPV Kit**  Now I’m going to read statements about using the HPV Screening Kit. Please tell me how much you agree or disagree with each of the statements.  *[Encourage full use of the Likert scale: “Okay, would you say you strongly agree, or just agree OR. Okay, would you say you strongly disagree, or just disagree”]*   1. The HPV kit instructions were easy to understand. *Would you say you …* 2. It was hard to use the HPV kit correctly. 3. The HPV test swab was easy to insert. 4. I am sure I sampled the right place. 5. I was bothered by the HPV kit. 6. I felt physically uncomfortable when using the HPV kit. 7. The HPV kit was painless. 8. Using the HPV kit was unpleasant. 9. Using the HPV kit was embarrassing. 10. I believe the HPV test result is correct. 11. I trust the HPV test result. 12. I felt in control of my health after using the HPV kit. 13. Using the HPV kit is a good thing to do for my health. 14. I would recommend the HPV kit to a friend. 15. I would use the HPV kit in the future. 16. I would prefer that a provider administer the Pap test in clinic than use the HPV kit at home. | **Quantitative Measure of Attitudes toward Home HPV Kit**  Now I’m going to read statements about using the HPV Screening Kit. Please tell me how much you agree or disagree with each of the statements.  *[Encourage full use of the Likert scale: “Okay, would you say you strongly agree, or just agree OR. Okay, would you say you strongly disagree, or just disagree”]*   1. The HPV kit instructions were easy to understand. *Would you say you …* 2. It was difficult to use the HPV kit correctly. 3. The HPV test swab was easy to insert. 4. I am sure I sampled the right place. 5. I was bothered by the HPV kit. 6. I felt physically uncomfortable when using the HPV kit. 7. The HPV kit was painless. 8. Using the HPV kit was unpleasant. 9. Using the HPV kit was embarrassing. 10. I believe the HPV test result is correct. 11. I trust the HPV test result. 12. By using the HPV kit, I felt like I was taking control of my health. 13. Using the HPV kit is a good thing to do for my health. 14. I would recommend the HPV kit to a friend. 15. I would use the HPV kit in the future. 16. I would prefer in-clinic screening to using the home HPV kit. |
| **Understanding of Home HPV Kit Test Results**  Now we are going to talk about receiving the results of your home HPV test and any follow up that was recommended.  1. How did you receive your results *[IF NEEDED: through the Kaiser Permanente web portal, formerly MyGroupHealth or call from your healthcare provider…]*? What information was conveyed in the email / during the call?  2. Did the [results message, healthcare provider call, etc.] recommend any next steps? What were they?  3. Thinking about the [results message, healthcare provider call, etc.], were there any words you didn’t understand or were confusing? What do you think other patients might not understand or might have questions about?  4. Did the [results message, healthcare provider, etc.] mention that (whether) you were HPV positive?  *If NO, continue with Questions 4a-4b. If YES, skip to Question 5.*  *Note: We expect here that the patient reports receiving an abnormal result. If she says the results are normal, we should not contradict her.*  a. After [receiving the results message, getting the phone call, etc.] and learning that your results were normal, how did it make you feel?  b. After [receiving the results message, getting the phone call, etc.], did you want more information? What sort of information?  *Skip to Future Use of Home HPV Kit*  5. Did it say anything about HPV types 16 or 18? What did it say about those HPV types?  6. Tell me in your own words, why is it important to know about HPV types 16 and 18?  7. After [receiving the results message, getting the phone call, etc.] how did it make you feel?  8. After [receiving the results message, getting the phone call, etc.], did you want more information? What sort of information?  **This probe was added mid-trial after adding a study website describing kit and what results mean:* The results message included a website with more information about home cervical cancer screening. Did you visit the website? Was that information helpful?  9. Was the [results message, healthcare provider call, etc.] helpful? *What* could the healthcare team (provider and staff) do *differently to improve* how results are delivered to you? | **Understanding of Home HPV Kit Test Results**  *(Goal: Characterize patient comprehension of the test results and satisfaction with information provided. See if results caused any negative affect or worry.)*  Now we are going to talk about receiving the results of your home HPV test and any follow up that your health care team recommended.   1. How did you receive your results – [*IF NEEDED: through the Kaiser Permanente web portal, or a call from a nurse…*]?    1. How soon after using the kit did you get the results?    2. Did you have any questions on how to interpret the test results and what to do next, if anything?    3. Did you receive your test results any other way? What other ways? 2. Did your healthcare team recommend any next steps?   *If YES:* What were they?   1. Thinking about when you got your results, were there any words you didn’t understand or that were confusing? What do you think other patients might not understand or might have questions about? 2. When you got your results, did they mention whether you were HPV positive?   *If YES, skip to Question 5.*  *If NO, continue and ask questions 4a-e.*  *Note: We expect here that the patient reports receiving an abnormal (HPV positive) result. If they say their results were normal or negative, we should not contradict them.*  After [*receiving the results message / getting the phone call / etc.*], how did you feel?   - 1. After receiving your results, did you want any more information?   *If YES, then ask*:   - - - - What sort of information?       - Did you refer back to the educational brochure we talked about earlier?   *If YES*: Was the brochure helpful?   - - - - Did you look anywhere else for information about HPV?   *If YES*: Where did you look? What did you find?   - - - - The results message sent through the MyKP portal includes a website with more information about home cervical cancer screening. Did you visit the website?   *If YES*: Was the website helpful?   - 1. What could your healthcare team (provider and staff) do differently to improve how the results of this home HPV kit are delivered to you?   2. We also want to understand how the home kit experience compares with your past screening experiences. Have you ever had abnormal Pap or positive HPV results?   *If YES*: Can you compare that experience to your home testing experience?  Skip to Closing Statement/Last Question  **For people who report having positive results** (Goal: characterize patient perceptions about results communication.)   1. Next, we want to ask you a bit about how you received your test results. Do you remember if your results mentioned anything about different types of HPV?   *If YES*: What did they say about HPV types?  *If NO*: Did they talk about HPV types 16 or 18?   1. After receiving your results, how did you feel? 2. After receiving your results, did you want any more information?   *If YES, then ask*:   - - - What sort of information?     - Did you refer back to the educational brochure we talked about earlier?   *If YES*: Was the brochure helpful?   - Did you look anywhere else for information about HPV?   *If YES*: Where did you look? What did you find?   - - - The results message sent through the MyKP portal includes a website with more information about home cervical cancer screening. Did you visit the website?   *If YES*: Was the website helpful? |
| **Interactions with Health Care Providers During Follow-Up Process**  You said you received a positive test result….  *[If at any point during the interview, the participant sounds upset or hesitates, remind them that their responses are confidential and will not be shared with their provider, however, their provider may receive overall feedback about the study that does not contain any personal identifiers.]*  1. What did you do after you received the result?  *If participant had any in-clinic follow-up, continue to Question 2;*  *If No Follow-up, continue to next section Reasons for Lack of Follow-up.*  2. When you met with your health care provider, what did they recommend? Please tell me about your experience with your provider.  3. What questions did you have for your provider?  4. What kind of information about HPV did your provider give you?  5. Did you look anywhere else for information about HPV? Where did you look and what did you find?  6. What aspects of the follow-up process have you found most difficult? Easy?  7. During the follow-up process, did you have any trouble scheduling a visit with your healthcare provider? Did you feel like you received follow-up care in a timely manner? | **Interactions with Health Care Providers During Follow-Up Process**  *(Goal: Characterize patient perceptions about the follow-up process.)*  You said you received a positive test result…  *[If at any point during the interview, the participant sounds upset or hesitates, remind them that they can take their time in answering and their responses are confidential and will not be shared with their provider.]*   1. Did anyone on your healthcare team recommend any follow-up (visits or tests)? 2. Did you discuss the recommended follow-up with your healthcare team? Can you tell me a little about that discussion? What questions did you have about the follow-up recommendations? 3. Was it easy to understand what the follow-up recommendations were for your positive HPV test?   ***If no*:** Do you have any suggestions on how your healthcare team could have better explained your recommendation?   1. Did you follow the recommendation of your healthcare team?   *If NO Follow-up****,*** *skip to next section Reasons for Lack of Follow up.*  *If YES continue below to Questions 5-9.*   1. During the follow-up process, did you have any trouble scheduling and completing follow-up care with your provider? Did you feel like you received your follow-up care in a timely manner? 2. What would have made the follow-up process easier for you? 3. What could your health care team do differently to support patients who need follow-up care? 4. What could your health care team do differently to improve how a home HPV kit is offered to you and others? 5. We also want to understand how the home kit experience compares with your past screening experiences. Have you ever had abnormal Pap or positive HPV results before?   *If YES*: Can you compare that experience to your home testing experience?  *Skip to Closing* |
| **Reasons for Lack of Follow-Up**  (*Used if participant states they did nothing after receiving abnormal HPV result)*  1. Why did you choose *not* to get the *[recommended procedure]*?  2. Did you discuss your decision with your provider? Please tell me about that discussion. What questions did you have for your provider?  3. Did you look anywhere else for information about HPV? Where did you look and what did you find? | **REASONS FOR LACK OF FOLLOW-UP**  *(Used if participant states they did nothing after receiving abnormal HPV result)*   1. Can you tell me why you didn’t get follow-up care? 2. What could your health care team do differently to support patients who need follow-up care? 3. What could your health care team do differently to improve how a home HPV kit is offered to you and others? 4. We also want to understand how the home kit experience compares with your past screening experiences. Have you ever had abnormal Pap or positive HPV results before?   *If YES*: Can you compare that experience to your home testing experience? |
| **Future Use of Home HPV Kits.**  1. Would you use the home HPV test kit again? Would you recommend it to your family and friends?  2. *What* could the healthcare team (provider and staff) do *differently to improve* how this new home HPV kit is offered to you and other patients? |  |
| **Closing**  *If interview is running short due to skip pattern (<15 minutes long), then ask:*  1. Before this home HPV kit result, have you ever had abnormal Pap or HPV results? *(If YES)* Tell me about that experience.  Those are all the questions we have for you today. Is there anything else you would like to share with the research team about the home HPV kit?  Thanks again for your time and for answering our questions. I will send you $50 as a thank you for your time. There is an informational on-line link included in the thank you letter. (*verify address*) Have a good (evening, day). | **CLOSING STATEMENT/LAST QUESTION:**  Those are all the questions we have for you today. Is there anything else you would like to share with the research team about the home HPV kit?  Thanks again for your time and for answering our questions. I will send you $50 as a thank you for your time. If you’d like more information on screening, we include a link to the CDC’s webpage in the thank you letter. *(verify address)* Have a good (evening, day). |
