# Supplementary material for: Improving Communication and Management Following a Positive Home HPV Self-Sampling Result: Comparing Intervention Strategies Between the HOME and STEP Trials
Source: Womens Health Rep (New Rochelle). 2025 Aug 25;6(1):771–81. doi: 10.1177/26884844251371093 (PMC12415163; doi:10.1177/26884844251371093)
Supplement: Supplementary Appendix A2 [file 26884844251371093_supp_appendixa2.docx]

**Appendix 2. COREQ Checklist**

| **Domain 1: Research team and reflexivity** | | |
| --- | --- | --- |
| **Personal Characteristics** | | |
| 1. Interviewer/facilitator | Which author/s conducted the interviews? | Page 3, 10 |
| 1. Credentials | What were the researcher’s credentials? | Page 1 |
| 1. Occupation | What was their occupation at the time of the study? | Page 1 |
| 1. Gender | Was the researcher male or female? | Page 4 |
| 1. Experience and training | What experience or training did the researcher have? | Page 1 |
| **Relationship with participants** | | |
| 1. Relationship established | Was a relationship established prior to study commencement? | See page 10 |
| 1. Participant knowledge of the interviewer | What did the participants know about the researcher? | See page 10 |
| 1. Interviewer characteristics | What characteristics were reported about the interviewer? | See page 1, 4, 11 |
| **Domain 2: Study design** | | |
| **Theoretical framework** | | |
| 1. Methodological orientation and theory | What methodological orientation was stated to underpin the study? | Page 11 |
| **Participant Selection** | | |
| 1. Sampling | How were the participants selected? | Page 9-10 |
| 1. Method of approach | How were participants approached? | Page 9-10 |
| 1. Sample size | How many participants were in the study? | Page 12 |
| 1. Non-participation | How many people refused to participate or dropped out? Reasons? | Page 12 |
| **Setting** | | |
| 1. Setting of data collection | Where was the data collected? | Page 9, 10 |
| 1. Presence of non-participants | Was anyone else present besides the participants and researchers? | Page 11 |
| 1. Description of sample | What are the important characteristics of the sample? | Page 9, 18 |
| **Data collection** | | |
| 1. Interview guide | Were questions, prompts, or guides provided by the authors? | Page 10, Appendix 1 |
| 1. Repeat interviews | Were repeat interviews carried out? | Page 11 |
| 1. Audio/visual recording | Did the research use audio or visual recording to collect the data? | Page 10 |
| 1. Field notes | Were field notes made during and/or after the interview? | Page 11 |
| 1. Duration | What was the duration of the interview? | Page 11 |
| 1. Data saturation | Was data saturation discussed? | Page 11 |
| 1. Transcripts returned | Were transcripts returned to participants for comment and/or correction? | Page 11 |
| **Domain 3: Analysis and findings** | | |
| **Data Analysis** | | |
| 1. Number of data coders | How many data coders coded the data? | Page 11 |
| 1. Description of the coding tree | Did authors provide a description of the coding tree? | Page 11 |
| 1. Derivation of themes | Were themes identified in advance or derived from data? | Page 11 |
| 1. Software | What software was used to manage the data? | Page 11 |
| 1. Participant checking | Did participants provide feedback on the findings? | Page 12 |
| **Reporting** | | |
| 1. Quotations presented | Were participant quotations presented to illustrate the themes/findings? Was each quotation identified? | Page 13-15, Table 3. |
| 1. Data and findings consistent | Was there consistency between data presented and the findings? | Page 13 |
| 1. Clarity of major themes | Were major themes clearly presented in findings? | Page 13-15 |
| 1. Clarity of minor themes | Is there a description of diverse cases or discussion of minor themes? | Page 13-15, 18 |
